# High-Throughput Multilocus Sequence Typing: Bringing Molecular Typing to the Next Level

**DOI:** 10.1371/journal.pone.0039630

**Published:** 2012-07-18

**Authors:** Stefan A. Boers, Wil A. van der Reijden, Ruud Jansen

**Affiliations:** Department of Molecular Biology, Regional Laboratory of Public Health, Haarlem, The Netherlands; Institut National de la Recherche Agronomique, France

## Abstract

Multilocus sequence typing (MLST) is a widely used system for typing microorganisms by sequence analysis of housekeeping genes. The main advantage of MLST in comparison to other typing techniques is the unambiguity and transferability of sequence data. However, a main disadvantage is the high cost of DNA sequencing. Here we introduce a high-throughput MLST (HiMLST) method that employs next-generation sequencing (NGS) technology (Roche 454), to generate large quantities of high-quality MLST data at low costs. The HiMLST protocol consists of two steps. In the first step MLST target genes are amplified by PCR in multi-well plates. During this PCR the amplicons of each bacterial isolate are provided with a unique DNA barcode, the multiplex identifier (MID). In the second step all amplicons are pooled and sequenced in a single NGS-run. The MLST profile of each individual isolate can be retrieved easily using its unique MID. With HiMLST we have profiled 575 isolates of *Legionella pneumophila*, *Staphylococcus aureus*, *Pseudomonas aeruginosa* and *Streptococcus pneumoniae* in mixed species HiMLST experiments. In conclusion, the introduction of HiMLST paves the way for a broad employment of the MLST as a high-quality and cost-effective method for typing microbial species.

## Introduction

Microbial typing techniques are greatly enhancing our insights into microbial population epidemiology and microbial diversity, and are widely used in diagnostic, genomic and pathogenesis-related microbiology research [1]. For example, in the field of clinical microbiology, phenotypic methods such as biochemical typing of Enterobacteriaceae [2] and serological typing of streptococci [3] are performed to differentiate within microbial species. Moreover, determining antimicrobial susceptibility of bacteria by antibiogram typing is routinely performed to guide infection treatment, but also serves as a first-line phenotypic method to identify possible nosocomial spread of bacterial pathogens. For more comprehensive studies of population structure, dynamics, and the molecular evolution of a certain bacterial species, phenotypic assays are not sufficient and the determination of a microbial genotype, rather than its phenotype, is a prerequisite [4]. For this, a variety of molecular typing techniques have been developed to compare the genotypes of microbial species. The utility of genotyping techniques depends upon the aim of the investigation as well as practical issues, e.g. discriminatory power, reproducibility, technical difficulty, time to result, throughput and costs.

Commonly used molecular typing methods are based on size fractionation of multiple DNA fragments, such as amplified fragment length polymorphism analysis (AFLP), pulsed-field gel electrophoresis (PFGE), and more recently multiple-locus VNTR analysis (MLVA). These methods have a good discriminatory power and can be scaled up to characterize a large number of bacterial isolates at low costs [5]–[7]. Nevertheless, as typing-project sizes grew with numerous bacterial isolates from different sampling locations and different sampling times, it became apparent that standardization of these methods between laboratories was hard to achieve and frustrated the development of reliable databases for epidemiological use [8]. To meet the strict requirements for database construction, methods were developed that are sequence-based. Examples are the single-locus sequence typing (SLST) methods, such as *spa* typing for *Staphylococcus aureus* [9], [10] and *emm* typing for *Streptococcus pyogenes* [3], [11], and the widely used multilocus sequence typing (MLST) schemes that are based on the sequence variations of multiple, often seven, housekeeping genes [12]. Due to the unambiguous character of DNA sequences, the MLST and other sequence-based typing methods are highly robust and the data achieved by different laboratories can be reliably compared using online databases [4], [13]. However, MLST protocols are relatively expensive to execute, mainly due to the laborious process of DNA sequencing by the Sanger technology, which is currently the most commonly used DNA sequencing technology [13], [14]. An approach to circumvent the high costs of sequencing for MLST is to analyse the PCR products by use of mass spectrometry [15], [16]. Such approaches are certainly useful for assigning genotypes to strains to already known clonal complexes, but the technology does not generate the actual DNA sequences of the MLST targets. The DNA sequences are necessary to identify new alleles of the MLST target genes and to determine phylogenetic relatedness of strains by comparing concatenated sequences.

In this study we introduce a cost-effective high-throughput MLST (HiMLST) approach that employs next-generation sequencing (NGS) to generate the sequence data. Since its introduction, NGS revolutionized sequence data generation in molecular biology. Several NGS-platforms have been developed to produce millions of high-quality bases at low costs within a single sequence run [14], [17]. For the HiMLST we employed the Genome Sequencer (GS) Junior (Roche, Almere, The Netherlands), that is able to generate up to 70.000 amplicon reads with average read lengths between 400–500 bases [18]. This long-read sequencing performance, in combination with a sample pooling strategy that uses “bar-coded” amplicons for parallel analysis of pooled samples, allows the generation of MLST profiles from multiple bacterial isolates in a single NGS-run. Earlier studies have shown that these bar-coding strategies are effective for massive parallel sequencing of pooled samples [19]–[22]. In this report, we demonstrate the successful parallel sequencing of MLST alleles that were amplified by PCR from 96 different bacterial isolates from four different species in single NGS-runs.

## Materials and Methods

### Bacterial Isolates

All bacterial isolates were obtained from bacterial cultures of the routine diagnostic laboratory at our institution. The Medical Ethical Research Committee decided that no review is required for this study on these bacterial isolates. A total of 575 bacterial isolates of four different species were selected for HiMLST. One hundred and five *Legionella pneumophila* isolates were obtained from clinical and environmental sources as part of a national outbreak detection programme in The Netherlands; 269 *Staphylococcus aureus* and 189 *Pseudomonas aeruginosa* isolates were obtained from patients at a burn centre; and twelve *Streptococcus pneumoniae* isolates were obtained from clinical blood samples from the routine diagnostics in our laboratory. The species of the bacterial isolates were determined using standard bacteriological techniques and matrix-assisted laser desorption/ionization time-of-flight (MALDI-TOF) mass spectrometry. The bacterial isolates are not traceable to individual patients, omitting the need for approval by an ethical committee.

### MLST Target Gene Amplification

DNA was extracted from bacterial cultures using the High Pure PCR Template Preparation Kit (Roche, Almere, The Netherlands) according to the manufacturer’s instructions. Housekeeping genes of interest were amplified by PCR using primers from standardized MLST schemes [23]–[26], but with universal tails at the 5′ end to allow the addition of 454 sequencing-specific nucleotides and isolate-specific multiplex identifiers (MIDs) in a second PCR round (table S1). Sequencing of PCR fragments by 454 sequencing technology requires certain adaptations to the DNA fragments. For the emulsion PCR (emPCR), the single-strand DNA fragments require binding sequences, called adapters, at the 5′ end for immobilization onto specifically designed DNA capture beads. There exist two kinds of such adapters, A and B, allowing the bidirectional sequencing of the target sequence. In addition, each fragment requires a four-nucleotide ‘key’ sequence that is recognized by the 454 software and indicates the starting point of a sequence read. This key is also used to differentiate reads from internal quality control sequences [27], [28]. PCRs were performed in 5 µl reaction volumes using the FastStart High Fidelity Reaction Kit (Roche) with the addition of 0.5 µM of each PCR primer. Resolight Dye (Roche) was added to measure DNA amplification in real-time on a LightCycler 480 instrument (Roche). The MLST target genes were amplified by PCR in 96 well plates, with the following cycling conditions: initial denaturation at 95°C for 2 minutes followed by 45 cycles of PCR, with cycling conditions of 30 seconds at 95°C, 30 seconds at 55°C, and 60 seconds at 72°C. The amplification and melting curves of the alleles were compared and alleles with aberrant curves were excluded from further analysis. The PCRs for *P. aeruginosa* required the addition of DMSO to a final concentration of 5% (v/v), due to the high GC-content of the *P. aeruginosa* housekeeping genes. After the PCRs, the amplicons were purified from unincorporated dNTPs, primers, primer dimers and salts using magnetic AMPure XP beads (Agencourt, Woerden, The Netherlands).

### Barcode Incorporation PCR

The purified MLST amplicons were re-amplified to incorporate 454 sequencing-specific nucleotides and isolate-specific MIDs. For this, we used fusion primers that target the universal tails, which were incorporated in the amplicons of the first PCR round (table S2). The fusion primers recognize the universal tails and consist of an MID and the 454 sequencing-specific key and A or B sequence. Again, all PCR reactions were performed with 5 µl reaction volumes using the FastStart High Fidelity Reaction Kit with the addition of 0.5 µM of each fusion primer and the Resolight Dye. The PCR reactions were performed on a LightCycler 480 instrument using a 96 well plate format, but under modified conditions: initial denaturation at 95°C for 2 minutes followed by 35 cycles of PCR, with cycling conditions of 30 seconds at 95°C, 30 seconds at 50°C and 60 seconds at 72°C. During the first 10 cycles of PCR, the annealing temperature was increased by 0.5°C per cycle to an annealing temperature of 55°C. Again, DMSO was added to a final concentration of 5% (v/v) to improve PCR efficiency during the re-amplification of the GC-rich *P. aeruginosa* housekeeping genes.

### Sample Pooling

Bar-coded amplicons were mixed in equimolar concentrations. The complete pool was purified by gel extraction using the QIAquick Gel Extraction Kit (Qiagen, Venlo, The Netherlands), followed by a second purification with magnetic AMPure XP beads. In preparation for 454 sequencing, the concentration of the pool of purified amplicons was measured using the Quant-iT PicoGreen dsDNA Assay Kit (Life Technologies Europe BV, Bleiswijk, The Netherlands) on a LightCycler 480 instrument and diluted to 1×10^9^ DNA molecules/µl.

### Emulsion PCR and 454 Sequencing

An emulsion-based clonal amplification (emPCR) was performed according to the manufacturer’s instructions as described in the emPCR Amplification Method Manual - Lib-A, revision June 2010 (Roche). The DNA sequencing was done using the GS Junior Titanium Sequencing Kit and the GS Junior Titanium PicoTiterPlate using the Sequencing Method Manual, revision June 2010 (Roche).

### Data Analysis

NGS-data were automatically processed using the ‘Full Processing Amplicon’ pipeline available through the Run Wizard on the GS Junior Attendant PC (Roche). This pipeline involves two steps that are leading to the conversion of the raw image data to base-called results. During the first step, active PicoTiterPlate regions are defined of which the raw signals for each nucleotide flow are extracted into ‘Composite Wells Format’ (CWF) files. Then during the second step, the CWF files are analyzed following a series of normalization, correction, and quality filtering algorithms. This step converts the remaining high-quality signals into ‘flowgrams’ for each read and generates base-calls with associated quality scores that are extracted into ‘Standard Flowgram Format’ (SFF) files. The read information in the SFF files serves as an input for the GS Reference Mapper software (Roche), which was used to identify the seven alleles from each individual isolate by their unique MID. Briefly, each read per isolate was mapped to reference allele variants, generating consensus sequences using the default parameters of the software. These parameters consist of a minimum accepted read length of 20 bp to be used in the mapping, and the following overlap detection settings: seed step, 12; seed length, 16; seed count, 1; hit-per-seed limit, 70; minimum overlap length, 40; minimum overlap identity, 90; alignment identity score, 2; alignment difference score, −3; repeat score threshold, 12. Finally, the allele variant numbers were obtained by performing queries in the species associated MLST databases, which are located on the Internet at http://www.hpa-bioinformatics.org.uk/legionella/legionella_sbt/php/sbt_homepage.php, http://www.mlst.net and http://pubmlst.org.

### Sanger Sequencing

To validate the employment of NGS for MLST and verification of newly found allele variants, 95 alleles were sequenced using standard techniques performed on the CEQ 8000 Genetic Analysis System platform (Beckman Coulter, Woerden, The Netherlands). These data were analyzed using Bionumerics version 5.10 (Applied Maths, Sint-Martens-Latem, Belgium). The 95 alleles included 12 new allele variants, six for *S. aureus*, five for *P. aeruginosa* and one for *S. pneumoniae*. The sequences of these new variants are deposited in GenBank with accession numbers JQ740582 to JQ740593.

### Statistical Analysis

The relation between the size of amplicons and the number of 454 sequence reads was assessed by use of linear regression analysis, with the number of reads as the dependent variable and the size as the independent variable (PASW Statistics, release 18.0, SPSS inc., Chicago Illinois).

**Figure 1:**
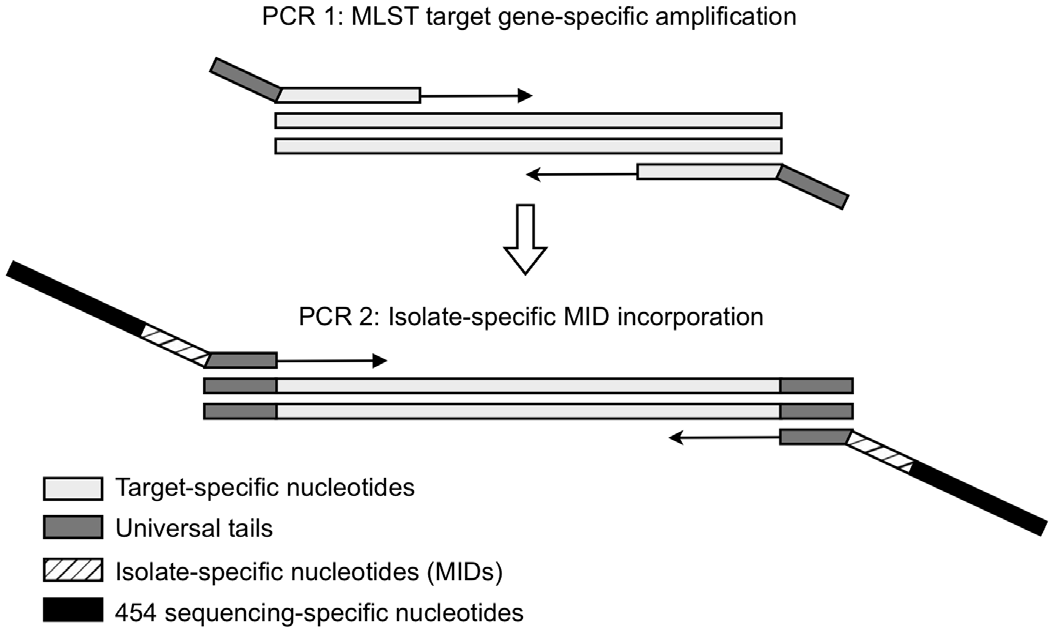
Two-Step PCR strategy for HiMLST. During the first PCR, the targeted MLST gene is amplified and universal tails are incorporated. During the second PCR, the amplicons of each isolate are provided with 454 sequencing-specific nucleotides and a unique DNA barcode, the multiplex identifier (MID).

## Results

**Figure 2:**
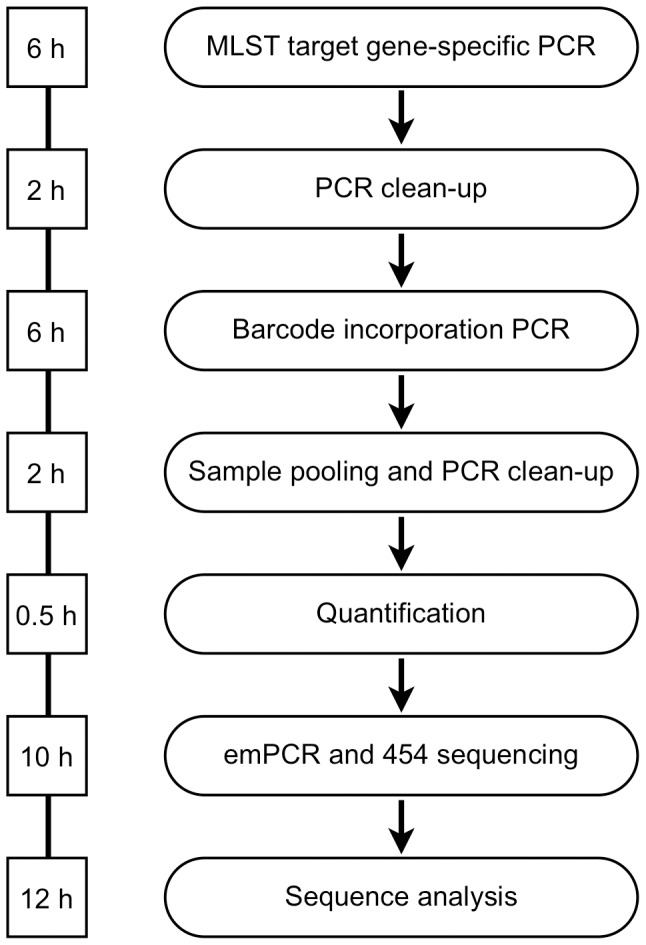
The workflow for profiling 96 bacterial isolates with HiMLST. MLST target genes are amplified by a two-step PCR in multi-well plates. These amplicons are pooled, quantified, clonally amplified by emulsion PCR and sequenced by NGS. The workflow ends with the ST profiling of the individual bacterial isolate. Figures in squares indicate the hands-on time for each individual step.

To employ 454 sequencing technology for HiMLST, the MLST amplicons require adaptations necessary for this technology. These adaptations consist of the incorporation of 454 sequencing-specific nucleotides at both ends of the amplicons and the incorporation of isolate-specific MID sequences. To introduce these adaptations, we have designed a two-step PCR approach that is depicted in figure 1. During the first round of PCR, the MLST target genes are amplified using primers that are specific for each gene of the bacterial species under study. These target-specific primers are provided at the 5′ end with universal tails. The forward and reverse target-specific primers carry different tails, which enables a bidirectional sequencing approach in the NGS. The PCR products of the first round were not directly applicable as a template for the second round of PCR. The tailed amplicons had to be purified with magnetic beads to remove unspecific PCR products of low molecular weight (results not shown). These unspecific PCR products were found to compete with the tailed amplicons in the second PCR round, resulting in poor yields of the desired products. In a second PCR round, the amplicons are re-amplified using a forward and reverse MID primer that targets the two universal tails of the target-specific primers. The MID primers are provided with the 454 sequencing-specific nucleotides and with a unique MID. The seven amplicons of each bacterial isolate are amplified in the second PCR with a set of forward and reverse MID primers to incorporate the specific MID for differentiation of the bacterial isolates of the HiMLST experiment. After the second PCR round, the amplicons of each MLST target gene were pooled, clonally amplified by emPCR and sequenced on a GS Junior platform (Roche) using standardized 454 sequencing protocols. Finally, the MLST profile of each individual isolate was generated using its unique MID (figure 2).

**Figure 3:**
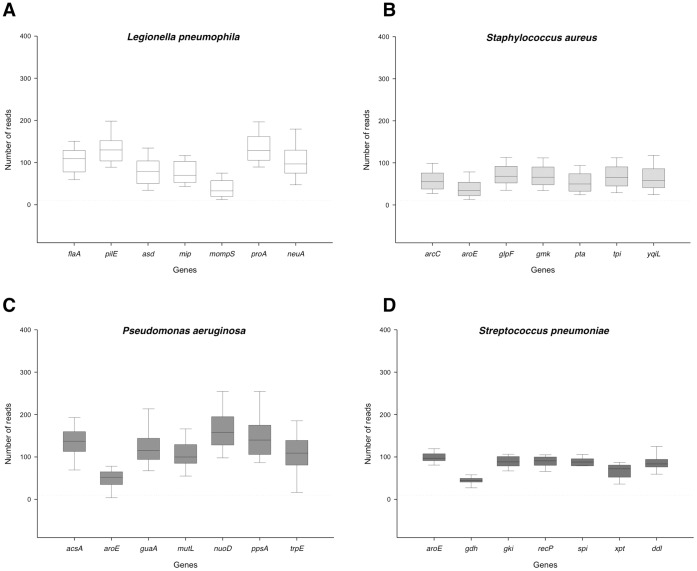
Number of reads per gene for *L. pneumophila*, *S. aureus*, *P. aeruginosa*, and *S. pneumoniae*. Box plots show median (middle line), 25^th^ percentile (lower box limit), 75^th^ percentile (upper box limit), 10^th^ percentile (lower whisker) and 90^th^ percentile (upper whisker) for number of reads per gene.

To prove the concept of HiMLST, we have profiled 575 isolates of *L. pneumophila*, *S. aureus*, *P. aeruginosa* and *S. pneumoniae* in NGS-runs with single and multiple species. In 97.2% of all bacterial isolates we were able to produce a full seven-allele profile, and hence a sequence type (ST). The failure to obtain a ST of nine of the 575 isolates could all be explained by the failure to get a PCR product of one of the alleles of the nine isolates, due to suboptimal PCR conditions. After repetition of the failed PCRs we were able to generate a full seven-allele profile for each of the isolates. This demonstrates that the manual processing of the PCRs is the most critical part of the protocol. In figure 3 we show the median of the number of sequence reads per gene for the four different bacterial species that were included in this study. This median ranged from 33 reads for *L. pneumophila mompS* genes till 158 reads per *P. aeruginosa nuoD* gene, leading to high-quality consensus sequences with general Phred-like quality values of 64.

**Figure 4:**
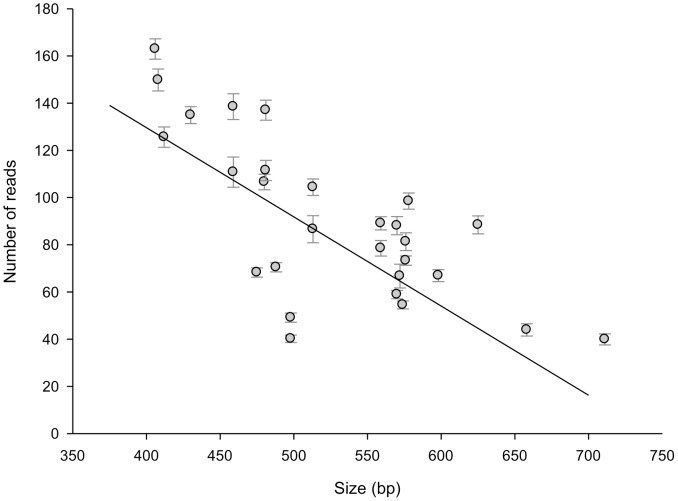
Relation between amplicon size and number of sequence reads. Mean number of reads (standard error of the mean) are plotted for the 28 different genes from the bacterial species *L. pneumophila, S. aureus, P. aeruginosa, and S. pneumoniae*. This relationship between size and number of reads is statistical significant (p-value <0.001, linear regression) and shows an R^2^ value of 0.23.

The established MLST schemes employ Sanger sequencing to obtain the data. In contrast to Sanger sequencing, the length of the amplicon is an important feature for NGS, since the efficiencies of the PCRs in the emulsion depend strongly on the lengths of the amplicons. Therefore, to obtain equal number of sequence reads for each allele from each species, it is important that in the HiMLST the amplicons are amplified with comparable efficiencies. To achieve this goal we had to adapt the PCR primers for some of the alleles resulting in reduced or increased amplicon sizes. Most notably this was done for *P. aeruginosa*, for which the standard MLST scheme relies on amplicons ranging from 800 till 1,000 bp in size [25]. These amplicons form the template for the sequencing reaction that uses a different primer set to sequence the 400 to 500 bp of interest. To be able to profile *P. aeruginosa* isolates with HiMLST, we amplified the internal gene fragments directly using the sequencing primers as described by Curren et al. [25]. The adaption of the amplicon sizes resulted in the successful sequencing by NGS of all alleles. Nevertheless, as shown in figure 3 there are still some differences between the sequence coverage of the alleles. It appeared that there is a relationship between the amplicon length and number of reads as shown in figure 4. This relationship is statistical significant (p-value <0.001, linear regression) and shows a R^2^ value of 0.23, indicating that 23% of the alleles show a good correlation between amplicon size and number of reads.

To validate the employment of NGS for MLST, we have compared a total of 95 alleles sequenced by both NGS and Sanger methodology and no discrepancies were found. We found twelve new alleles among the species *S. pneumoniae, P. aeruginosa* and *S. aureus* and all were confirmed by Sanger sequencing. The eleven new alleles of *P. aeruginosa* and *S. aureus* of the NGS sequencing were accepted by the database curators and added to the respective databases. The single *S. pneumoniae* allele was submitted as a Sanger sequencing file.

## Discussion

In this study, we introduce a high-throughput MLST strategy that allows the genotyping of large numbers of bacterial isolates at low costs. The HiMLST employs the 454 sequencing technology to sequence the MLST alleles from 96 bacterial isolates in a single NGS-run. The costs of 454 sequencing are relatively high, expressed per mega base pair, when compared to other NGS-platforms [17]. However, the long reads of 400 to 500 bp that are provided with the 454-platform are essential for the HiMLST protocol. Thus far, only the 454 sequencing technology can provide these long reads, but it is expected that in the near future other NGS-platforms such as the IonTorrent also can provide reads up to 400 bp. The introduction of IonTorrent technology in HiMLST may result in a significant cost reduction of instrumentation and reagents. Alternative platforms that produce at a low price short sequences of approximately 100 bp are no alternative for HiMLST, since it will be cumbersome to sequence simultaneously the long MLST amplicons from a large numbers of strains. In addition, the large NGS-systems, such as the Illumina-Solexa, ABI SOLiD and the GS 454 FLX have a capacity that is often too large for the average MLST projects of several hundred of isolates. The moderate capacity and the relatively low price of a NGS-run on the 454 GS Junior platform gives the system a low threshold to start a new experiment and allows HiMLST runs with several dozen to several hundred of bacterial isolates.

The design of the two-step HiMLST protocol with the tailed primers to incorporate the 454 sequencing-specific key, A and B sequences and isolate-specific MIDs is a flexible and economical way to realize a high-throughput MLST. The incorporation of the 454 sequencing-specific nucleotides and MID sequences into an amplicon can be done by using tailed primers carrying these additional sequences at the 5′ end. However, the addition of the 454 sequencing-specific nucleotides and MIDs in a single PCR round requires a large number of long primers for a single NGS-run. For example, in an NGS-run with 96 individual bacterial isolates of a certain species of which 7 alleles are amplified, one would need 96×14 = 1344 primers. For a HiMLST-run with an other bacterial species, again the same number of primers has to be synthesized. The synthesis of such vast numbers of large primers of approximately 60 bp will be very costly, making this approach uneconomical. This problem was also encountered by Singh and co-workers who recently introduced the MLST-seq method that uses hairpin primers to amplify different target loci and simultaneously incorporates 454 sequencing-specific nucleotides and MIDs [19]. The advantage of this approach is that species-specific primers can be combined with universal primers that carry the 454 sequencing-specific nucleotides and MIDs, without the need to synthesize large numbers of lengthy primers. However, this approach resulted in an uneven sequence coverage of the loci and consequently a large number of missed targets [19]. To circumvent these problems, we have designed an efficient two-step PCR approach that is depicted in figure 1. The two-step PCR approach requires for a single NGS-run of 96 isolates with 7 alleles only 14 gene-specific primers and 2×96 = 192 universal MID primers, each carrying an unique MID and the 454 specific sequences. Not only is the total number of primers much lower (206 versus 1344), but the main advantage of this approach is that for each new bacterial species to be subjected to HiMLST, only 14 new target-specific primers have to be synthesized that can be used with the universal set of MID primers.

Currently, the 454 sequence technology produces reads with an average length between 400–500 bases [18]. The sequences needed for most existing MLST protocols are over 500 bp in length and therefore a bidirectional read of the amplicons is necessary to obtain a full sequence. Because of this, read coverage is a very important issue to ensure the correct identification of nucleotides. With HiMLST we have profiled 575 bacterial isolates of four different species with a median of sequence coverage ranging from 33 to 158 reads per allele. Smith et al. have shown that a read coverage of 10 to 15 reads is accurate for mutational profiling in yeast using 454 sequencing [29]. The sequences of the presented HiMLST-runs have a double coverage of that number of reads, indicating that with the current protocol the capacity of the HiMLST can be doubled to 192 bacterial isolates and retain the high-quality of the sequence data.

A balanced read coverage was an important issue in the development of the HiMLST. One of the factors that influences the number of reads turned out to be the size of the amplicon. In figure 4 we show the statistical significant correlation between amplicon size and sequence coverage in 23% of the alleles. Obviously, factors as GC-content and intrinsic properties of the amplicon PCRs, play a role in the read numbers of the alleles. The easiest way to obtain a more balanced distribution of reads is to adapt the volumes of amplicons in the sample pooling prior to the emPCR. The optimal volume needs to be tested empirically for each individual allele. Using this strategy, we were able to gain a more balanced distribution of reads by adapting the volumes of the *L. pneumophila mompS, S. aureus aroE,* and *P. aeruginosa aroE* housekeeping genes during the sample pooling (data not shown).

Another important factor that influences the quality of the sequencing data is the presence of stretches of identical bases in the amplicon sequences. Such homo-polymeric stretches are abundant in the MLST target genes of *S. pneumoniae* and are also common in the MLST targets of *Escherichia coli* and *Salmonella enterica*. Pyrosequencing, which is the technology for the 454-platform has an inherently low performance at reading long stretches of homo-polynucleotides. This well-known technical limitation of pyrosequencing results in an underestimation of the number of bases in a homo-polynucleotide stretch [17], [18] and hence to mistakes and low-quality scores for individual bases in the stretches. Fortunately, the HiMLST with a coverage of 50 reads per amplicon will have ample reads in which the full length stretch are correctly annotated and the contig can be corrected accordingly. In addition, the HiMLST suffers less from the wrongly annotated stretches than *de novo* sequencing, because the absence of a single base in a stretch implicates that the allele of the analyzed housekeeping gene suffers from a very unlikely frame-shift mutation. Nevertheless, the presence of homo-polymeric stretches requires constant attention and the manual correction of contigs is a laborious and unwanted process.

The introduction of the HiMLST paves the way to use the robust typing by MLST on a large scale by eliminating its major draw back; the high costs. For example, in our laboratory the HiMLST reduces the costs of MLST per bacterial isolate by a factor of ten compared to the formerly used Sanger sequencing. The costs of a HiMLST typing with the current protocol, including labor and reagents are 38 US dollars (30 Euro) per bacterial strain. Despite this major reduction in costs, the costs per bacterial isolate can even be further reduced by implementing a more efficient workflow and by increasing the number of isolates in a NGS-run. Specification of the costs of NGS showed that two-thirds of costs consist of reagents and disposables and one-third of the costs can be accounted to labour. As shown in figure 2, the total hands on time for performing a single HiMLST experiment takes up 38.5 hours. However, two major time-consuming steps are noticed which can be further optimized. First, amplicon preparation is accountable for 14 hours (36%). This workload can be reduced significantly by implementing pipetting robots to prepare the large number of PCRs. Moreover, with pipetting robots it is possible to reduce the PCR reaction volumes, which reduces the cost of PCR reagents. Secondly, sequence analysis of the different alleles requires 12 hours (31%). This can be reduced drastically when using optimized software that can handle HiMLST data with direct linkage towards online MLST databases. Apart of these optimizations of the workflow, the capacity of the HiMLST can be doubled to 192 isolates per NGS-run retaining the high-quality of the sequences, leading to a potential twenty-fold reduction of costs compared to Sanger sequencing.

The HiMLST can be adapted to the existing MLST schemes for a number of bacterial species as shown in this study and the HiMLST can be easily expanded to other bacterial species. The reduction of sequencing costs also allows for an increase in the discriminatory power of the MLST by expanding of the number of genes of the MLST protocol. It might even be feasible to implement the universal ribosomal MLST typing scheme for bacterial species in which up to 53 genes are included [30].

MLST is also a commonly used typing method for diploid microorganisms, such as fungi, yeasts and protozoa. Similar to the bacterial MLST protocols, the MLST protocols for these diploid organisms can also be adapted to HiMLST, but the HiMLST also has the potential to improve MLST schemes for typing of diploid organisms. Diploid organisms carry two alleles for each gene. With the Sanger sequencing technology it is impossible to separate the sequences of both alleles and the resulting sequence is a mixture of both allele sequences, limiting the use of MLST for these organisms [31]. With NGS sequencing it is expected that both alleles can be analyzed separately resulting in the two haplotypes of the gene. This improvement in the typing for this group of organisms will increase the typing potential of MLST.

To conclude, the HiMLST approach opens new perspectives for the large-scale application of the robust MLST technique. The HiMLST results in a substantial reduction of labour and costs compared to the traditional Sanger sequencing, thus paving the way for MLST to become an attractive and feasible technique for molecular typing of both haploid and diploid microorganisms.

## Supporting Information

Table S1 **MLST target gene-specific primers used in this study.** Nucleotides in black represent the gene-specific part and universal tails are shown in red or blue.
(PDF)

Table S2 **454 sequencing fusion primers used in this study.** Nucleotides in black and pink represent respectively 454 sequencing-specific adapter nucleotides and the key sequence; MID sequences are shown in green; and universal tails are depicted in red or blue.
(PDF)
