# Supplementary material for: High-Throughput Multilocus Sequence Typing: Bringing Molecular Typing to the Next Level
Source: PLoS One. 2012 Jul 18;7(7):e39630. doi: 10.1371/journal.pone.0039630 (PMC3399827; doi:10.1371/journal.pone.0039630)
Supplement: Table S1 — MLST target gene-specific primers used in this study. Nucleotides in black represent the gene-specific part and universal tails are shown in red or blue. (PDF) [file pone.0039630.s001.pdf]

Table S1. MLST target gene-specific primers used in this study. Nucleotides in black represent the gene-specific part and universal tails are shown in red or blue.

Locus	Direction	Primer sequence (5' to 3')	Reference
L. pneumophila flaA	Forward	GACACTATAGTATGCGTGAGCTTTCCGTTC	Unpublished [1]
	Reverse	CACTATAGGGCCATTAATCGTTAAGTTGTAGG	Unpublished [1]
L. pneumophila pilE	Forward	GACACTATAGCACAATCGGATGGAACACAAACTA	[23]
	Reverse	CACTATAGGGCTGGCGCACTCGGTATCT	[23]
L. pneumophila asd	Forward	GACACTATAGCCCTAATTGCTCTACCATTCAGATG	[23]
	Reverse	CACTATAGGGCGAATGTTATCTGCGACTATCCAC	[23]
L. pneumophila mip	Forward	GACACTATAGGCTGCAACCGATGCCAC	[23]
	Reverse	CACTATAGGGCATATGCAAGACCTGAGGGAAC	[23]
L. pneumophila mompS	Forward	GACACTATAGTTGACCATGAGTGGGATTG	Unpublished [1]
	Reverse	CACTATAGGGTGGATAAATTATCCAGCCGGACTTC	[23]
L. pneumophila proA	Forward	GACACTATAGGATCGCCAATGCAATTAG	[23]
	Reverse	CACTATAGGGACCATAACATCAAAAGCC	[23]
L. pneumophila neuA	Forward	GACACTATAGCCGTTCAATATGGGGCTTCAG	[23]
	Reverse	CACTATAGGGCGATGTCGATGGATTCACTAATAC	[23]
S. aureus arcC	Forward	GACACTATAGTTGATTCACCAGCGCGTATTGTC	[24]
	Reverse	CACTATAGGGAGGTATCTGCTTCAATCAGCG	[24]
S. aureus aroE	Forward	GACACTATAGATCGGAAATCCTATTTCACATTC	[24]
	Reverse	CACTATAGGGTGTTGTATTAATAACGATATC	[24]
S. aureus glpF	Forward	GACACTATAGCTAGGAACTGCAATCTTAATCC	[24]
	Reverse	CACTATAGGGTGGTAAAATCGCATGTGCAATTC	[24] [2]
S. aureus gmk	Forward	GACACTATAGATCGTTTTATCAGGACCATC	[24] [2]
	Reverse	CACTATAGGGTCATTAACTACAACGTAATCGTA	[24]
S. aureus pta	Forward	GACACTATAGGTTAAAATCGTATTACCTGAAGG	[24]
	Reverse	CACTATAGGGACCCTTTTGTTGAAAAGCTTAA	[24]
S. aureus tpi	Forward	GACACTATAGTCGTTCATTCTGAACGTCGTGAA	[24]
	Reverse	CACTATAGGGTTTGCACCTTCTAACAATTGTAC	[24]
S. aureus yqiL	Forward	GACACTATAGCAGCATACAGGACACCTATTGGC	[24]
	Reverse	CACTATAGGGCGTTGAGGAATCGATACTGGAAC	[24]
P. aeruginosa acsA	Forward	GACACTATAGGCCACACCTACATCGTCTAT	[25]
	Reverse	CACTATAGGGAGGTTGCCGAGGTTGTCCAC	[25]
P. aeruginosa aroE	Forward	GACACTATAGATGTCACCGTGCCGTTCAAG	[25]
	Reverse	CACTATAGGGTGAAGGCAGTCGGTTCCTTG	[25]
P. aeruginosa guaA	Forward	GACACTATAGAGGTCGGTTCCTCCAAGGTC	[25]
	Reverse	CACTATAGGGACGTTGTGGTGCGACTTGA	[25]
P. aeruginosa mutL	Forward	GACACTATAGAGAAGACCGAGTTCGACCAT	[25]
	Reverse	CACTATAGGGTGCCATAGAGGAAGTCAT	[25]
P. aeruginosa nuoD	Forward	GACACTATAGACGGCGAGAACGAGGACTAC	[25]
	Reverse	CACTATAGGGTGGCGGTCGGTGAAGGTGAA	[25]
P. aeruginosa ppsA	Forward	GACACTATAGGTGACGACGGCAAGCTGTA	[25]
	Reverse	CACTATAGGGTATCGCCTTCGGCACAGGA	[25]
P. aeruginosa trpE	Forward	GACACTATAGTTCAACTTCGGCGACTTCCA	[25]
	Reverse	CACTATAGGGTGTCCATGTTGCCGTTCC	[25]
S. pneumoniae aroE	Forward	GACACTATAGCCTTTGAGGCGACAGC	[26]
	Reverse	CACTATAGGGTGCAGTTCARAAACATWTTCTAA	[26]
S. pneumoniae gdh	Forward	GACACTATAGATGGACAAACCAGCNAGYTT	[26]
	Reverse	CACTATAGGGCTTGAGGTCCCATRCTNCC	[26]
S. pneumoniae gki	Forward	GACACTATAGGCATTGGAATGGGATCACC	[26]
	Reverse	CACTATAGGGTCTCCCGCAGCTGACAC	[26]
S. pneumoniae recP	Forward	GACACTATAGCCAACTCAGGTCATCCAGG	[26]
	Reverse	CACTATAGGGTGCAACCGTAGCATTGTAAC	[26]
S. pneumoniae spi	Forward	GACACTATAGTTATTCCTCCTGATTCTGTC	[26]
	Reverse	CACTATAGGGTGATTGGCCAGAAGCGGAA	[26]
S. pneumoniae xpt	Forward	GACACTATAGTTATTAGAAGAGCGCATCCT	[26]
	Reverse	CACTATAGGGAGATCTGCCTCCTTAAATAC	[26]
S. pneumoniae ddl	Forward	GACACTATAGTGCYCAAGTTCCTTATGTGG	[26]
	Reverse	CACTATAGGGCACTGGGTRAAACCWGGCAT	[26]

[1]: Primers adopted from M13 variant of the nested sequence-based typing (SBT) protocol for epidemiological typing of *L. pneumophila* directly from clinical samples. Prepared by Massimo Mentasti and Norman Fry - version 1.0, 9th October 2009.

[2]: Primers modified by Deurenberg et al. *Antimicrob Agents Chemother*, 49(10): 4263-4271.
