# Supplementary material for: High-Throughput Multilocus Sequence Typing: Bringing Molecular Typing to the Next Level
Source: PLoS One. 2012 Jul 18;7(7):e39630. doi: 10.1371/journal.pone.0039630 (PMC3399827; doi:10.1371/journal.pone.0039630)
Supplement: Table S2 — 454 sequencing fusion primers used in this study. Nucleotides in black and pink represent respectively 454 sequencing-specific adapter nucleotides and the key sequence; MID sequences are shown in green; and universal tails are depicted in red or blue. (PDF) [file pone.0039630.s002.pdf]

Table S2. 454 sequencing fusion primers used in this study. Nucleotides in black and pink represent respectively 454 sequencing-specific adapter nucleotides and the key sequence; MID sequences are shown in green; and universal tails are depicted in red or blue.

Fusion primer	Primer sequence (5' to 3')
PrimA-RL013-FORW	CGTATCGCCTCCCTCGCGCCATCAGAGACTCGACGTGACACTATAG
PrimB-RL013-REV	CTATGCGCCTTGCCAGCCCGCTCAGAGACTCGACGTCACTATAGGG
PrimA-RL014-FORW	CGTATCGCCTCCCTCGCGCCATCAGAGTACGAGAGTGACACTATAG
PrimB-RL014-REV	CTATGCGCCTTGCCAGCCCGCTCAGAGTACGAGAGTCACTATAGGG
PrimA-RL015-FORW	CGTATCGCCTCCCTCGCGCCATCAGAGTACTACTATGACACTATAG
PrimB-RL015-REV	CTATGCGCCTTGCCAGCCCGCTCAGAGTACTACTATCACTATAGGG
PrimA-RL016-FORW	CGTATCGCCTCCCTCGCGCCATCAGAGTAGACGTCTGACACTATAG
PrimB-RL016-REV	CTATGCGCCTTGCCAGCCCGCTCAGAGTAGACGTCTCACTATAGGG
PrimA-RL017-FORW	CGTATCGCCTCCCTCGCGCCATCAGAGTCGTACACTGACACTATAG
PrimB-RL017-REV	CTATGCGCCTTGCCAGCCCGCTCAGAGTCGTACACTCACTATAGGG
PrimA-RL018-FORW	CGTATCGCCTCCCTCGCGCCATCAGAGTGTAGTAGTGACACTATAG
PrimB-RL018-REV	CTATGCGCCTTGCCAGCCCGCTCAGAGTGTAGTAGTCACTATAGGG
PrimA-RL019-FORW	CGTATCGCCTCCCTCGCGCCATCAGATAGTATACGTGACACTATAG
PrimB-RL019-REV	CTATGCGCCTTGCCAGCCCGCTCAGATAGTATACGTCACTATAGGG
PrimA-RL020-FORW	CGTATCGCCTCCCTCGCGCCATCAGCAGTACGTACTGACACTATAG
PrimB-RL020-REV	CTATGCGCCTTGCCAGCCCGCTCAGCAGTACGTACTCACTATAGGG
PrimA-RL021-FORW	CGTATCGCCTCCCTCGCGCCATCAGCGACGACGCGTGACACTATAG
PrimB-RL021-REV	CTATGCGCCTTGCCAGCCCGCTCAGCGACGACGCGTCACTATAGGG
PrimA-RL022-FORW	CGTATCGCCTCCCTCGCGCCATCAGCGACGAGTACTGACACTATAG
PrimB-RL022-REV	CTATGCGCCTTGCCAGCCCGCTCAGCGACGAGTACTCACTATAGGG
PrimA-RL023-FORW	CGTATCGCCTCCCTCGCGCCATCAGCGATACTACGTGACACTATAG
PrimB-RL023-REV	CTATGCGCCTTGCCAGCCCGCTCAGCGATACTACGTCACTATAGGG
PrimA-RL024-FORW	CGTATCGCCTCCCTCGCGCCATCAGCGTACGTCGATGACACTATAG
PrimB-RL024-REV	CTATGCGCCTTGCCAGCCCGCTCAGCGTACGTCGATCACTATAGGG
PrimA-RL025-FORW	CGTATCGCCTCCCTCGCGCCATCAGCTACTCGTAGTGACACTATAG
PrimB-RL025-REV	CTATGCGCCTTGCCAGCCCGCTCAGCTACTCGTAGTCACTATAGGG
PrimA-RL026-FORW	CGTATCGCCTCCCTCGCGCCATCAGGTACAGTACGTGACACTATAG
PrimB-RL026-REV	CTATGCGCCTTGCCAGCCCGCTCAGGTACAGTACGTCACTATAGGG
PrimA-RL027-FORW	CGTATCGCCTCCCTCGCGCCATCAGGTCGTACGTATGACACTATAG
PrimB-RL027-REV	CTATGCGCCTTGCCAGCCCGCTCAGGTCGTACGTATCACTATAGGG
PrimA-RL028-FORW	CGTATCGCCTCCCTCGCGCCATCAGGTGTACGACGTGACACTATAG
PrimB-RL028-REV	CTATGCGCCTTGCCAGCCCGCTCAGGTGTACGACGTCACTATAGGG
PrimA-RL029-FORW	CGTATCGCCTCCCTCGCGCCATCAGACACAGTGAGTGACACTATAG
PrimB-RL029-REV	CTATGCGCCTTGCCAGCCCGCTCAGACACAGTGAGTCACTATAGGG
PrimA-RL030-FORW	CGTATCGCCTCCCTCGCGCCATCAGACACTCATACTGACACTATAG
PrimB-RL030-REV	CTATGCGCCTTGCCAGCCCGCTCAGACACTCATACTCACTATAGGG
PrimA-RL031-FORW	CGTATCGCCTCCCTCGCGCCATCAGACAGACAGCGTGACACTATAG
PrimB-RL031-REV	CTATGCGCCTTGCCAGCCCGCTCAGACAGACAGCGTCACTATAGGG
PrimA-RL032-FORW	CGTATCGCCTCCCTCGCGCCATCAGACAGACTATATGACACTATAG
PrimB-RL032-REV	CTATGCGCCTTGCCAGCCCGCTCAGACAGACTATATCACTATAGGG
PrimA-RL033-FORW	CGTATCGCCTCCCTCGCGCCATCAGACAGAGACTCTGACACTATAG
PrimB-RL033-REV	CTATGCGCCTTGCCAGCCCGCTCAGACAGAGACTCTCACTATAGGG
PrimA-RL034-FORW	CGTATCGCCTCCCTCGCGCCATCAGACAGCTCGTGTGACACTATAG
PrimB-RL034-REV	CTATGCGCCTTGCCAGCCCGCTCAGACAGCTCGTGTCACTATAGGG
PrimA-RL035-FORW	CGTATCGCCTCCCTCGCGCCATCAGACAGTGTCGATGACACTATAG
PrimB-RL035-REV	CTATGCGCCTTGCCAGCCCGCTCAGACAGTGTCGATCACTATAGGG
PrimA-RL036-FORW	CGTATCGCCTCCCTCGCGCCATCAGACGAGCGCGCTGACACTATAG
PrimB-RL036-REV	CTATGCGCCTTGCCAGCCCGCTCAGACGAGCGCGCTCACTATAGGG
PrimA-RL037-FORW	CGTATCGCCTCCCTCGCGCCATCAGACGATGAGTGTGACACTATAG
PrimB-RL037-REV	CTATGCGCCTTGCCAGCCCGCTCAGACGATGAGTGTCACTATAGGG
PrimA-RL038-FORW	CGTATCGCCTCCCTCGCGCCATCAGACGCGAGAGATGACACTATAG
PrimB-RL038-REV	CTATGCGCCTTGCCAGCCCGCTCAGACGCGAGAGATCACTATAGGG
PrimA-RL039-FORW	CGTATCGCCTCCCTCGCGCCATCAGACGCTCTCTCTGACACTATAG
PrimB-RL039-REV	CTATGCGCCTTGCCAGCCCGCTCAGACGCTCTCTCTCACTATAGGG
PrimA-RL040-FORW	CGTATCGCCTCCCTCGCGCCATCAGACGTCGCTGATGACACTATAG
PrimB-RL040-REV	CTATGCGCCTTGCCAGCCCGCTCAGACGTCGCTGATCACTATAGGG
PrimA-RL041-FORW	CGTATCGCCTCCCTCGCGCCATCAGACGTCTAGCATGACACTATAG
PrimB-RL041-REV	CTATGCGCCTTGCCAGCCCGCTCAGACGTCTAGCATCACTATAGGG
PrimA-RL042-FORW	CGTATCGCCTCCCTCGCGCCATCAGACTAGTGATATGACACTATAG
PrimB-RL042-REV	CTATGCGCCTTGCCAGCCCGCTCAGACTAGTGATATCACTATAGGG
PrimA-RL043-FORW	CGTATCGCCTCCCTCGCGCCATCAGACTCACACTGTGACACTATAG
PrimB-RL043-REV	CTATGCGCCTTGCCAGCCCGCTCAGACTCACACTGTCACTATAGGG
PrimA-RL044-FORW	CGTATCGCCTCCCTCGCGCCATCAGACTCACTAGCTGACACTATAG
PrimB-RL044-REV	CTATGCGCCTTGCCAGCCCGCTCAGACTCACTAGCTCACTATAGGG

PrimA-RL045-FORW	CGTATCGCCTCCCTCGCGCCATCAGACTCTATATATGACACTATAG
PrimB-RL045-REV	CTATGCGCCTTGCCAGCCCGCTCAGACTCTATATATCACTATAGGG
PrimA-RL046-FORW	CGTATCGCCTCCCTCGCGCCATCAGACTGATCTCGTGACACTATAG
PrimB-RL046-REV	CTATGCGCCTTGCCAGCCCGCTCAGACTGATCTCGTCACTATAGGG
PrimA-RL047-FORW	CGTATCGCCTCCCTCGCGCCATCAGACTGCTGTACTGACACTATAG
PrimB-RL047-REV	CTATGCGCCTTGCCAGCCCGCTCAGACTGCTGTACTCACTATAGGG
PrimA-RL048-FORW	CGTATCGCCTCCCTCGCGCCATCAGACTGTAGCGCTGACACTATAG
PrimB-RL048-REV	CTATGCGCCTTGCCAGCCCGCTCAGACTGTAGCGCTCACTATAGGG
PrimA-RL049-FORW	CGTATCGCCTCCCTCGCGCCATCAGAGACACTCACTGACACTATAG
PrimB-RL049-REV	CTATGCGCCTTGCCAGCCCGCTCAGAGACACTCACTCACTATAGGG
PrimA-RL050-FORW	CGTATCGCCTCCCTCGCGCCATCAGAGACATATAGTGACACTATAG
PrimB-RL050-REV	CTATGCGCCTTGCCAGCCCGCTCAGAGACATATAGTCACTATAGGG
PrimA-RL051-FORW	CGTATCGCCTCCCTCGCGCCATCAGAGACGTGATCTGACACTATAG
PrimB-RL051-REV	CTATGCGCCTTGCCAGCCCGCTCAGAGACGTGATCTCACTATAGGG
PrimA-RL052-FORW	CGTATCGCCTCCCTCGCGCCATCAGAGAGTACAGATGACACTATAG
PrimB-RL052-REV	CTATGCGCCTTGCCAGCCCGCTCAGAGAGTACAGATCACTATAGGG
PrimA-RL053-FORW	CGTATCGCCTCCCTCGCGCCATCAGAGAGTATCTCTGACACTATAG
PrimB-RL053-REV	CTATGCGCCTTGCCAGCCCGCTCAGAGAGTATCTCTCACTATAGGG
PrimA-RL054-FORW	CGTATCGCCTCCCTCGCGCCATCAGAGATACGCTGTGACACTATAG
PrimB-RL054-REV	CTATGCGCCTTGCCAGCCCGCTCAGAGATACGCTGTCACTATAGGG
PrimA-RL055-FORW	CGTATCGCCTCCCTCGCGCCATCAGAGATCTAGTCTGACACTATAG
PrimB-RL055-REV	CTATGCGCCTTGCCAGCCCGCTCAGAGATCTAGTCTCACTATAGGG
PrimA-RL056-FORW	CGTATCGCCTCCCTCGCGCCATCAGAGCAGCGTAGTGACACTATAG
PrimB-RL056-REV	CTATGCGCCTTGCCAGCCCGCTCAGAGCAGCGTAGTCACTATAGGG
PrimA-RL057-FORW	CGTATCGCCTCCCTCGCGCCATCAGAGCGCACGAGTGACACTATAG
PrimB-RL057-REV	CTATGCGCCTTGCCAGCCCGCTCAGAGCGCACGAGTCACTATAGGG
PrimA-RL058-FORW	CGTATCGCCTCCCTCGCGCCATCAGAGCGTGTGCGTGACACTATAG
PrimB-RL058-REV	CTATGCGCCTTGCCAGCCCGCTCAGAGCGTGTGCGTCACTATAGGG
PrimA-RL059-FORW	CGTATCGCCTCCCTCGCGCCATCAGAGCTAGATACTGACACTATAG
PrimB-RL059-REV	CTATGCGCCTTGCCAGCCCGCTCAGAGCTAGATACTCACTATAGGG
PrimA-RL060-FORW	CGTATCGCCTCCCTCGCGCCATCAGAGCTGTCGACTGACACTATAG
PrimB-RL060-REV	CTATGCGCCTTGCCAGCCCGCTCAGAGCTGTCGACTCACTATAGGG
PrimA-RL061-FORW	CGTATCGCCTCCCTCGCGCCATCAGAGTATGCACGTGACACTATAG
PrimB-RL061-REV	CTATGCGCCTTGCCAGCCCGCTCAGAGTATGCACGTCACTATAGGG
PrimA-RL062-FORW	CGTATCGCCTCCCTCGCGCCATCAGAGTCGCGCTATGACACTATAG
PrimB-RL062-REV	CTATGCGCCTTGCCAGCCCGCTCAGAGTCGCGCTATCACTATAGGG
PrimA-RL063-FORW	CGTATCGCCTCCCTCGCGCCATCAGAGTCTGTCTGTGACACTATAG
PrimB-RL063-REV	CTATGCGCCTTGCCAGCCCGCTCAGAGTCTGTCTGTCACTATAGGG
PrimA-RL064-FORW	CGTATCGCCTCCCTCGCGCCATCAGATACACACGATGACACTATAG
PrimB-RL064-REV	CTATGCGCCTTGCCAGCCCGCTCAGATACACACGATCACTATAGGG
PrimA-RL065-FORW	CGTATCGCCTCCCTCGCGCCATCAGATACGCGTGCTGACACTATAG
PrimB-RL065-REV	CTATGCGCCTTGCCAGCCCGCTCAGATACGCGTGCTCACTATAGGG
PrimA-RL066-FORW	CGTATCGCCTCCCTCGCGCCATCAGATACTAGCACTGACACTATAG
PrimB-RL066-REV	CTATGCGCCTTGCCAGCCCGCTCAGATACTAGCACTCACTATAGGG
PrimA-RL067-FORW	CGTATCGCCTCCCTCGCGCCATCAGATAGAGCTAGTGACACTATAG
PrimB-RL067-REV	CTATGCGCCTTGCCAGCCCGCTCAGATAGAGCTAGTCACTATAGGG
PrimA-RL068-FORW	CGTATCGCCTCCCTCGCGCCATCAGATATAGAGTATGACACTATAG
PrimB-RL068-REV	CTATGCGCCTTGCCAGCCCGCTCAGATATAGAGTATCACTATAGGG
PrimA-RL069-FORW	CGTATCGCCTCCCTCGCGCCATCAGATCGCTCACGTGACACTATAG
PrimB-RL069-REV	CTATGCGCCTTGCCAGCCCGCTCAGATCGCTCACGTCACTATAGGG
PrimA-RL070-FORW	CGTATCGCCTCCCTCGCGCCATCAGATCGTCAGTCTGACACTATAG
PrimB-RL070-REV	CTATGCGCCTTGCCAGCCCGCTCAGATCGTCAGTCTCACTATAGGG
PrimA-RL071-FORW	CGTATCGCCTCCCTCGCGCCATCAGATCTCTCGTATGACACTATAG
PrimB-RL071-REV	CTATGCGCCTTGCCAGCCCGCTCAGATCTCTCGTATCACTATAGGG
PrimA-RL072-FORW	CGTATCGCCTCCCTCGCGCCATCAGATCTGAGACGTGACACTATAG
PrimB-RL072-REV	CTATGCGCCTTGCCAGCCCGCTCAGATCTGAGACGTCACTATAGGG
PrimA-RL073-FORW	CGTATCGCCTCCCTCGCGCCATCAGATGCTACGTCTGACACTATAG
PrimB-RL073-REV	CTATGCGCCTTGCCAGCCCGCTCAGATGCTACGTCTCACTATAGGG
PrimA-RL074-FORW	CGTATCGCCTCCCTCGCGCCATCAGATGTGACTACTGACACTATAG
PrimB-RL074-REV	CTATGCGCCTTGCCAGCCCGCTCAGATGTGACTACTCACTATAGGG
PrimA-RL075-FORW	CGTATCGCCTCCCTCGCGCCATCAGCACGAGACAGTGACACTATAG
PrimB-RL075-REV	CTATGCGCCTTGCCAGCCCGCTCAGCACGAGACAGTCACTATAGGG
PrimA-RL076-FORW	CGTATCGCCTCCCTCGCGCCATCAGCACGCGAGTCTGACACTATAG
PrimB-RL076-REV	CTATGCGCCTTGCCAGCCCGCTCAGCACGCGAGTCTCACTATAGGG
PrimA-RL077-FORW	CGTATCGCCTCCCTCGCGCCATCAGCACGCTACGATGACACTATAG
PrimB-RL077-REV	CTATGCGCCTTGCCAGCCCGCTCAGCACGCTACGATCACTATAGGG
PrimA-RL078-FORW	CGTATCGCCTCCCTCGCGCCATCAGCACGTGTATATGACACTATAG
PrimB-RL078-REV	CTATGCGCCTTGCCAGCCCGCTCAGCACGTGTATATCACTATAGGG
PrimA-RL079-FORW	CGTATCGCCTCCCTCGCGCCATCAGCACTACGATGTGACACTATAG
PrimB-RL079-REV	CTATGCGCCTTGCCAGCCCGCTCAGCACTACGATGTCACTATAGGG
PrimA-RL080-FORW	CGTATCGCCTCCCTCGCGCCATCAGCACTATACTCTGACACTATAG
PrimB-RL080-REV	CTATGCGCCTTGCCAGCCCGCTCAGCACTATACTCTCACTATAGGG

PrimA-RL081-FORW	CGTATCGCCTCCCTCGCGCCATCAGCAGCGTACTGTGACACTATAG
PrimB-RL081-REV	CTATGCGCCTTGCCAGCCCGCTCAGCAGCGTACTGTCACTATAGGG
PrimA-RL082-FORW	CGTATCGCCTCCCTCGCGCCATCAGCAGTCTCTAGTGACACTATAG
PrimB-RL082-REV	CTATGCGCCTTGCCAGCCCGCTCAGCAGTCTCTAGTCACTATAGGG
PrimA-RL083-FORW	CGTATCGCCTCCCTCGCGCCATCAGCATAGTCGCGTGACACTATAG
PrimB-RL083-REV	CTATGCGCCTTGCCAGCCCGCTCAGCATAGTCGCGTCACTATAGGG
PrimA-RL084-FORW	CGTATCGCCTCCCTCGCGCCATCAGCGAGACACTATGACACTATAG
PrimB-RL084-REV	CTATGCGCCTTGCCAGCCCGCTCAGCGAGACACTATCACTATAGGG
PrimA-RL085-FORW	CGTATCGCCTCCCTCGCGCCATCAGCGAGAGTGTGTGACACTATAG
PrimB-RL085-REV	CTATGCGCCTTGCCAGCCCGCTCAGCGAGAGTGTGTCACTATAGGG
PrimA-RL086-FORW	CGTATCGCCTCCCTCGCGCCATCAGCGAGTCATCGTGACACTATAG
PrimB-RL086-REV	CTATGCGCCTTGCCAGCCCGCTCAGCGAGTCATCGTCACTATAGGG
PrimA-RL087-FORW	CGTATCGCCTCCCTCGCGCCATCAGCGATCGTATATGACACTATAG
PrimB-RL087-REV	CTATGCGCCTTGCCAGCCCGCTCAGCGATCGTATATCACTATAGGG
PrimA-RL088-FORW	CGTATCGCCTCCCTCGCGCCATCAGCGCAGTACGCTGACACTATAG
PrimB-RL088-REV	CTATGCGCCTTGCCAGCCCGCTCAGCGCAGTACGCTCACTATAGGG
PrimA-RL089-FORW	CGTATCGCCTCCCTCGCGCCATCAGCGCGATCGTATGACACTATAG
PrimB-RL089-REV	CTATGCGCCTTGCCAGCCCGCTCAGCGCGATCGTATCACTATAGGG
PrimA-RL090-FORW	CGTATCGCCTCCCTCGCGCCATCAGCGCGCTATACTGACACTATAG
PrimB-RL090-REV	CTATGCGCCTTGCCAGCCCGCTCAGCGCGCTATACTCACTATAGGG
PrimA-RL091-FORW	CGTATCGCCTCCCTCGCGCCATCAGCGTACAGATATGACACTATAG
PrimB-RL091-REV	CTATGCGCCTTGCCAGCCCGCTCAGCGTACAGATATCACTATAGGG
PrimA-RL092-FORW	CGTATCGCCTCCCTCGCGCCATCAGCGTAGCTCTCTGACACTATAG
PrimB-RL092-REV	CTATGCGCCTTGCCAGCCCGCTCAGCGTAGCTCTCTCACTATAGGG
PrimA-RL093-FORW	CGTATCGCCTCCCTCGCGCCATCAGCGTATAGTGCTGACACTATAG
PrimB-RL093-REV	CTATGCGCCTTGCCAGCCCGCTCAGCGTATAGTGCTCACTATAGGG
PrimA-RL094-FORW	CGTATCGCCTCCCTCGCGCCATCAGCGTCAGCGACTGACACTATAG
PrimB-RL094-REV	CTATGCGCCTTGCCAGCCCGCTCAGCGTCAGCGACTCACTATAGGG
PrimA-RL095-FORW	CGTATCGCCTCCCTCGCGCCATCAGCGTCGCAGTGTGACACTATAG
PrimB-RL095-REV	CTATGCGCCTTGCCAGCCCGCTCAGCGTCGCAGTGTCACTATAGGG
PrimA-RL096-FORW	CGTATCGCCTCCCTCGCGCCATCAGCGTCTCACGATGACACTATAG
PrimB-RL096-REV	CTATGCGCCTTGCCAGCCCGCTCAGCGTCTCACGATCACTATAGGG
PrimA-RL097-FORW	CGTATCGCCTCCCTCGCGCCATCAGCGTGACTCAGTGACACTATAG
PrimB-RL097-REV	CTATGCGCCTTGCCAGCCCGCTCAGCGTGACTCAGTCACTATAGGG
PrimA-RL098-FORW	CGTATCGCCTCCCTCGCGCCATCAGCTACACGCTCTGACACTATAG
PrimB-RL098-REV	CTATGCGCCTTGCCAGCCCGCTCAGCTACACGCTCTCACTATAGGG
PrimA-RL099-FORW	CGTATCGCCTCCCTCGCGCCATCAGCTACGATATGTGACACTATAG
PrimB-RL099-REV	CTATGCGCCTTGCCAGCCCGCTCAGCTACGATATGTCACTATAGGG
PrimA-RL100-FORW	CGTATCGCCTCCCTCGCGCCATCAGCTAGACAGACTGACACTATAG
PrimB-RL100-REV	CTATGCGCCTTGCCAGCCCGCTCAGCTAGACAGACTCACTATAGGG
PrimA-RL101-FORW	CGTATCGCCTCCCTCGCGCCATCAGCTAGTACTCATGACACTATAG
PrimB-RL101-REV	CTATGCGCCTTGCCAGCCCGCTCAGCTAGTACTCATCACTATAGGG
PrimA-RL102-FORW	CGTATCGCCTCCCTCGCGCCATCAGCTATATGTCGTGACACTATAG
PrimB-RL102-REV	CTATGCGCCTTGCCAGCCCGCTCAGCTATATGTCGTCACTATAGGG
PrimA-RL103-FORW	CGTATCGCCTCCCTCGCGCCATCAGCTATCGACACTGACACTATAG
PrimB-RL103-REV	CTATGCGCCTTGCCAGCCCGCTCAGCTATCGACACTCACTATAGGG
PrimA-RL104-FORW	CGTATCGCCTCCCTCGCGCCATCAGCTATGTAGAGTGACACTATAG
PrimB-RL104-REV	CTATGCGCCTTGCCAGCCCGCTCAGCTATGTAGAGTCACTATAGGG
PrimA-RL105-FORW	CGTATCGCCTCCCTCGCGCCATCAGCTCACGTACATGACACTATAG
PrimB-RL105-REV	CTATGCGCCTTGCCAGCCCGCTCAGCTCACGTACATCACTATAGGG
PrimA-RL106-FORW	CGTATCGCCTCCCTCGCGCCATCAGCTCGAGTCTCTGACACTATAG
PrimB-RL106-REV	CTATGCGCCTTGCCAGCCCGCTCAGCTCGAGTCTCTCACTATAGGG
PrimA-RL107-FORW	CGTATCGCCTCCCTCGCGCCATCAGCTCGTCGAGATGACACTATAG
PrimB-RL107-REV	CTATGCGCCTTGCCAGCCCGCTCAGCTCGTCGAGATCACTATAGGG
PrimA-RL108-FORW	CGTATCGCCTCCCTCGCGCCATCAGCTCTACAGCGTGACACTATAG
PrimB-RL108-REV	CTATGCGCCTTGCCAGCCCGCTCAGCTCTACAGCGTCACTATAGGG
